# 

**DOI:** 10.1038/sj.bjc.6600404

**Published:** 2002-01-02


British Journal of Cancer (2002) 86(Suppl 1), S13–S33
© 2002 Cancer Research UK All rights reserved 0007–0920/02 $25.00
www.bjcancer.com

### 1.1
### INDIUM[111] PENTETREOTIDE SCINTIGRAPHY IN THE DIAGNOSIS AND MANAGEMENT OF NON-IODINE AVID CARCINOMA OF THE THYROID.

J Christian*[1], G Cook[1], C Harmer[1]
Royal Marsden Hospital, Downs Road, Sutton, United Kingdom, SM2 5PT[1]

The treatment of well-differentiated thyroid cancer has been a success of modern medicine with the use of radioiodine ($I^{131}$). Follicular and papillary carcinoma subtypes may occasionally however be non-iodine avid. The medullary carcinoma and the rare Hurthle cell carcinoma (HCC) subtypes are characterised by being non-iodine avid. All thyroid tumour types regularly express somatostatin receptors subtypes sstr1, sstr3, sstr4, and sstr5[1]. Octreotide, an analogue of somatostatin, can be combined with a radioactive isotope, (eg Indium$^{111}$ Pentetreotide) to visualize tumours with high concentrations of somatostatin receptors. We assessed 12 patients with histologically proven thyroid carcinoma including 10 patients with HCC (8 with metastatic disease), 1 with non-iodine avid metastatic papillary carcinoma and 1 with locally recurrent medullary carcinoma. In the case of metastatic disease the diagnosis was made using conventional radiology (CR), ie plain x-ray, ultrasound and computerised tomography. All had prospective scintigraphy using In$^{111}$Pentetreotide. Out of 8 patients with metastatic HCC, 6 had In$^{111}$ Pentetreotide positive scans. The sites of metastases were correlated with results from conventional radiology and shown in Table 1. The two patients with localised HCC underwent In$^{111}$Pentetreotide scintigraphy as a post operative staging assessment and images showed no evidence of residual disease. Both remaining patients with medullary or papillary carcinoma had positive In$^{111}$ Pentetreotide scintigraphy.

In conclusion In$^{111}$Pentetreotide imaging for patients with non-iodine avid carcinoma of the thyroid is a useful tool, both for the diagnosis and staging of metastases and potentially for treatment purposes. One patient with metastatic HCC to bone and a positive octreotide scan has now been treated with Yttrium-labelled octreotide.

Table 1. The distribution of metastases within the group of patients with metastatic Hurthle Cell Carcinoma undergoing scintigraphy with In$^{111}$Pentetreotide

| Patient No. | Site of metastases with conventional radiology | Iodine Uptake | Octreotide Uptake | Octreotide and CR correlation |
|---|---|---|---|---|
| 1 | Hilar LNs, lung, brain | Negative | Positive | CR better |
| 2 | Bone, LNs, liver, adrenal | Not tested | Negative | N/A |
| 3 | Cervical LNs, lung | Negative | Positive | Concordant |
| 4 | Bony skull base | Negative | Negative | N/A |
| 5 | Residual thyroid disease | Not tested | Positive | Concordant |
| 6 | Mediastinal LNs, lung | Negative | Positive | CR better |
| 7 | Recurrent neck disease | Negative | Positive | Concordant |
| 8 | Bone | Negative | Positive | Octreotide better |

LNs = lymph nodes N/A = not applicable

1. E.B. Forssell-Aronsson et al, 2000, J Nucl Med **41**(4): p 636

### 1.2
### *IN VIVO* EVALUATION OF [$^{18}$F]FLUOROETANIDAZOLE FOR IMAGING TUMOUR HYPOXIA WITH POSITRON EMISSION TOMOGRAPHY (PET)

Henryk Barthel*, Gavin Brown, Hanna Wilson, David R. Collingridge, Pat M. Price, Eric O. Aboagye.
PET Oncology Group, MRC Clinical Sciences Centre, Faculty of Medicine, Imperial College of Science, Technology and Medicine, Hammersmith Hospital Campus, London, United Kingdom

**Background**: Establishing a procedure to noninvasively diagnose tumour hypoxia is still a challenge for the medical imaging community. [$^{18}$F]Fluoroetanidazole ([$^{18}$F]FETA) is a new generation radiolabelled 2-nitroimidazole, which has been designed to overcome the problems connected with the first generation PET hypoxia marker, such as slow and insufficient uptake. So far, this new radiotracer has not been tested systematically *in vivo*. Therefore, this present study was initiated.

**Methods**: *Ex vivo* biodistribution studies were performed 1 hour after administration of [$^{18}$F]FETA into RIF-1 mouse fibrosarcoma, EMT6 mouse mammary carcinoma and HT1080-26.6 human fibrosarcoma bearing mice. These studies were paralleled by *in vivo* dynamic PET imaging using a small animal scanner. In addition, in the case of RIF-1 tumours hypoxia was modulated by carbogen (decrease) and hydralazine (increase) administration. In the aforementioned tumour models, independent data on the degree of hypoxia were obtained by measuring (1) the radiobiological hypoxic fraction by clonogenic assays and (2) the tumour oxygenation using Oxylite probes (Oxford Optronix Ltd, UK).

**Results**: The table shows the results of the clonogenic assays, Oxylite measurements and [$^{18}$F]FETA biodistribution studies in the different tumour models:

| Tumour | Radiobiological hypoxic fraction [%] | $pO_2$ < 2.5 mm Hg [%] | [$^{18}$F]FETA uptake [%ID/g] |
|---|---|---|---|
| RIF-1 | 60.4 ± 0.6 | 49 | 5.8 ± 0.8 |
| HT1080-26.6 | 68.1 ± 0.2 | 57 | 6.0 ± 0.5 |
| EMT6 | 78.1 ± 4.5 | 84 | 7.2 ± 0.6 |

In the RIF-1 tumour bearing mice, carbogen / hydralazine administration resulted in a decrease / increase in the relative frequency of $pO_2$ values < 2.5 mm Hg in the Oxylite measurements (40 vs. 67 %; $p < 0.001$); this was in concordance with a decrease / increase in the tumour [$^{18}$F]FETA uptake (4.9 ± 0.2 vs. 8.1 ± 0.6 %ID/g; $p = 0.007$). The PET imaging data with [$^{18}$F]FETA were in agreement with the biodistribution studies, but in addition provided dynamic information.

**Conclusion**: These initial data suggest that [$^{18}$F]FETA could by a useful PET marker for imaging tumour hypoxia. The technique would be suitable not only for primary diagnosis, but also to monitor bioreductive and antiangiogenic therapies.

*Supported by a grant awarded by the Leopoldina Society (Halle, Germany) and administered through the German Education and Research Ministry (BMBF-LPD 9901/8-22)

### 1.3
### A RADIATION DOSE RESPONSE EXISTS FOR RADIOIMMUNOTHERAPY (RIT) OF B-CELL LYMPHOMA ONLY IN THE PRESENCE OF SIGNALLING MONOCLONAL ANTIBODY (mAb)

Y. Du, J. Honeychurch, P.W.M Johnson, M. J. Glennie, T.M. Illidge* Cancer Sciences Division, Southampton University School of Medicine, Southampton, UK. SO16 6YD

Radioimmunotherapy (RIT) has emerged as an effective therapy for B cell lymphomas. There is however uncertainty whether myeloablative or lower (non-myeloablative) radiation doses should be used. We have investigated the relative contributions made by mAb and targeted radiation to tumour clearance *in vivo* and whether a radiation dose response exists for radioimmunotherapy. The biodistribution, organ dosimetry and therapeutic effect of a panel of $^{131}$I labeled B-cell specific mAbs (anti-CD19, anti-CD22, anti-MHCII and anti-Idiotype [Id]) was assessed in two syngeneic murine B-cell lymphoma models ($BCL_1$ and A31). Anti-MHCII mAb was estimated to deliver the highest total body and tumour-bearing organ radiation dose (18 Gy per 18.5 MBq of conjugated $^{131}$I to spleen). In contrast anti-Id, anti-CD19 and anti-CD22 mAbs delivered significantly less dose to tumour in spleen (3.4 Gy, 5.1 Gy, 5 Gy respectively).

Tumour protection was assessed using the same reagents in the lymphoma models following inoculation of $10^6$ tumour cells. Treatments were given after the development of tumour 10 or 15 days later. Anti-Id and anti-CD19 but not anti-MHCII mAb demonstrated modest therapeutic efficacy as unlabelled mAb in the $BCL_1$ model. When conjugated with 18.5 MBq of $^{131}$I, all three radioimmunoconjugates provided similar levels of tumour protection of around 20 days more than controls. For radiolabelled anti-MHCII mAb no dose response was seen and therapy was the same with either 9.25 MBq or 18.5 MBq of $^{131}$I. In contrast when unlabelled anti-Id or anti-CD19 was added to radiolabelled anti-MHC II a clear radiation dose response was observed.

More than 55 days improvement in tumour protection was seen for 9.25 MBq anti-MHC II + anti-Id and over 80% of animals were cured when treated with the combination of 18.5 MBq anti-MHC II + anti-Id. A similar radiation dose response was seen in the A31 tumour model with 100 % cures using the combination of a radiolabelled mAb and the signalling anti-Id mAb. In conclusion we have demonstrated for the first time in vivo that a radiation dose response exists for RIT of B-cell lymphoma and that the long term clearance of tumour is composed of two components, namely targeted radiation and mAb induced cytotoxicity, for which cell surface signalling appears important. These findings have important implications for the clinical application of RIT for B cell lymphomas. This work was funded by Cancer Research UK

### 1.4
### CAN BOLD MRI BE USED AS A NON-INVASIVE METHOD TO INVESTIGATE TUMOUR VESSEL MATURITY USING ANGIOTENSIN II?

*KJ Lankester, GM Tozer, SA Hill, RJ Maxwell
Gray Cancer Institute, Mount Vernon Hospital, Northwood, Middx, HA6 2JL.

**Introduction:** Animal tumour models show a variable response to vascular targeting agents, such as combretastatin, which may relate to variable levels of blood vessel maturity. Staining of tumour sections for alpha smooth muscle actin (αSMA) can be used to assess vessel maturity. Neeman et al.[1] proposed the use of Blood Oxygen Level Dependent (BOLD) MRI during periods of air, 5% $CO_2$ and carbogen (5%$CO_2$, 95%$O_2$) breathing as a non-invasive assessment of vessel maturity. BOLD MRI is sensitive to changes in blood flow and/or blood oxygenation due to the intrinsic MR contrast provided by deoxyhaemoglobin (deoxyHb). The gas-breathing protocol assumes that hypercapnia will result in vasodilatation of mature blood vessels (but not immature vessels) and consequent increase in MR signal intensity (positive BOLD effect). However, many tumour blood vessels are likely to be maximally dilated, so using a vasoconstrictor instead of $CO_2$ might be more effective. The purpose of this study was to determine whether using a peripheral vasoconstrictor such as angiotensin II (AT) in combination with BOLD-MRI can be used to estimate the degree of tumour vessel maturity.
**Methods:** The human colon carcinoma, HT29 and the CaNT murine tumour were used in mice when approx 350mg. Mice were anaesthetised before being placed in a 4.7T Varian MR system. BOLD MRI involved multi-gradient-echo imaging - 8 echoes; TE 5-40ms, TR 117ms, 4 averages, spatial resolution 0.16x0.31x1mm, time resolution 60s. 8 images were obtained before treatment, a further 16 images during AT infusion (2μg/kg/min for 16 min, i.v.) then another 8 images during recovery. Tumours were excised and stained for αSMA.
**Results:** There were no obvious responses to AT in 6/6 HT29 tumours. However, in 2/5 CaNT tumours there was a sustained 4% increase in signal intensity following the start of AT and a decrease following the end of the infusion. Preliminary analysis of the αSMA staining confirmed that CaNT has minimal staining and HT29 is more highly stained.
**Discussion:** CaNT has a large response to combretastatin and a minimal amount of αSMA staining whereas HT29 has the reverse. A positive BOLD response to AT seen in 2/5 CaNT is compatible with the absence of a direct vasoconstricting effect on tumour vessels resulting in increased tumour perfusion due to the AT-induced rise in systemic blood pressure i.e. it implies a lack of mature blood vessels. Tumours with more mature vessels could show no BOLD response (as here with HT29) or a negative BOLD response if there is an overall reduction in tumour perfusion due to vasoconstriction. The pattern of BOLD response is consistent with combretastatin sensitivity and αSMA staining in these tumour lines. BOLD has the advantage of providing intrinsic contrast, but other MR measures of changes in blood flow or volume might give a more direct measure of vessel maturity.
**Reference**: 1. Neeman M et al. Magn Reson Med 2001;45:887.
**Funding:** Marie Curie Translational Research Fund; Cancer Research UK; Gray Cancer Institute.

### 1.5
### CARBOGEN AND NICOTINAMIDE SELECTIVELY INCREASE CHEMOTHERAPY DELIVERY AND BLOOD FLOW TO LIVER METASTASIS

Nishi Gupta, Azeem Saleem, Safiye Osman, Peter Hoskin*, Patricia M Price, Eric O Aboagye
Cancer Research UK, PET Oncology Group, Hammersmith Hospital, Du Cane Rd, London W12 ONN, UK
*Mount Vernon Hospital, Northwood, Middlesex, UK

Aim: A combination of carbogen (95% oxygen and 5 % carbon dioxide) and nicotinamide (C/N) has been shown to improve drug delivery and blood flow (BF) in tumours in animals (McSheehy, 1998, Cancer Res, 58, 1185). We have measured the effect of carbogen and nicotinamide on 5-FU delivery and perfusion in tumour in man, *in vivo*, using positron emission tomography (PET).
Method: 6 patients, M/F 3/3, mean age 64 years (range 52-78) receiving 1[st] line De Gramont chemotherapy for metastatic colorectal cancer were studied. All patients underwent dynamic PET scanning with ($H_2[O^{15}]$) prior to and 5-[$^{18}$F]FU concurrently with their 5-FU bolus on 2 cycles of chemotherapy. The second PET scan was performed 2-3 hours after ingestion of nicotinamide (60mg/kg), with carbogen breathing commencing 5 minutes prior to scan start and for 5 minutes after (10 minutes in total). 5-FU delivery was calculated using standard uptake value at 3 minutes ($SUV_3$) and BF (ml blood/min/ml tissue) and vascular volume of distribution (VD) calculated using a 1- tissue compartment model.
Results: In total, 5-FU, $SUV_3$ blood flow and VD in 10 liver metastasis (> 3 cm) and the spleen as an example of normal tissue, were evaluated in the 6 patients:

| | Liver metastasis | Spleen |
|---|---|---|
| Mean 5 FU $SUV_3$ pre C/N | $6.277x10^{-5}$ | $1.264x10^{-4}$ |
| Mean 5 FU $SUV_3$ post C/N | $8.271x10^{-5}$ | $1.202x10^{-4}$ |
| Mean VD pre C/N | 0.78 | 0.89 |
| Mean VD post C/N | 0.72 | 0.91 |
| Mean BF pre C/N | 0.2292 | 1.880 |
| Mean BF post C/N | 0.3369 | 1.823 |

There was a significant increase in 5-FU $SUV_3$ (p=0.0137) and blood flow (p=0.0039) to liver metastasis following C/N, with no change in VD. Splenic BF, 5-FU $SUV_3$ and VD showed no significant changes after C/N. Arterial blood gas analysis showed a mean increase in $pO_2$ from 93mmHg (range 77-118) to 278mmHg (range 213-319) after 7.5-10 minutes of carbogen breathing, and returned to normal 5 minutes after cessation of carbogen inhalation. $pCO_2$ and pH remained unchanged throughout.
Discussion: C/N selectively increases BF and 5-FU delivery in human tumours. This system provides a model for the assessment of new agents postulated to improve chemotherapy delivery to human tumours.
This work is supported by a core MRC grant and CRC grants (SP2193/0401, SP2193/0202).

### 1.6
### HOW BEST TO DETERMINE RELAPSE/PROGRESSION IN OVARIAN CANCER

I.M. Hennig*[1], L. Clarkson[2], A. Sanderson[2], G.D. Hall[1], T.J. Perren[1], J.A. Spencer[2]
Cancer Research UK Clinical Centre [1] & Department of Radiology[2], St. James's University Hospital Leeds, LS9 7TF, UK

**Aim**: To determine best practice to diagnose progressive disease in patients with ovarian cancer.
**Introduction**: From 1991 to 1997, 97 patients from Leeds were randomised to maintenance interferon or no further treatment following surgery and/or induction chemotherapy within a national phase III clinical trial. Clinical follow-up with CA125 levels occurred every two, three, four months during the first, second and third year after randomisation, and six monthly thereafter. Routine CT was performed every six months for the first two years.
**Methods**: Retrospective review comparing clinical data, CA125 results and CT scans. This study was approved by the United Leeds Teaching Hospital Ethics Committee.
**Results**: 84 patients (pts) had complete clinical and radiological data. 2 pts developed second malignancies and were excluded from the final analysis.

 © 2002 Cancer Research UK

73 pts were CA125 positive (MP) at some stage and 9 were CA125 negative (MN) ***All patients:*** Progressive disease (PD) occurred in 75 of all 82 pts. This was detectable clinically in 27 pts and by ↑ CA125 in 53. 60 of the 70 MP pts (85.7%) were determined to have progressed by clinical findings and/or rising CA125. ***Complete remission:*** 49 pts were in complete remission (CR) at trial entry, 7 remain disease free. Relapse was detectable clinically in 11 pts. 30 had rising CA125 levels prior to or at relapse. 3 pts with normal markers were diagnosed with PD clinically prior to CT. Disease progression was confirmed by CT in 40 pts (one pt died prior to CT, one had no CT evidence of PD). Median time from elevation of CA125 to radiological PD was 44 days. 9 of 42 pts were therefore clinically well with a normal CA125 at the time of CT defined PD. ***Residual disease***: 33 pts had residual disease at trial entry (2 pts had only ↑ CA125). 13 had persistent elevation of CA125, 20 had a normal CA125. All 33 progressed (31 with confirmation on CT). PD was detectable in 17 pts by clinical examination and 25 had ↑ CA125. 2 pts with normal markers had clinical PD prior to CT. Median time from elevation of CA125 to radiological PD was 50 days. 2 of the 33 pts were clinically well with a normal CA125 at the time of CT defined PD. ***Marker negative:*** 5 of the 9 MN pts achieved a radiological complete remission, 1 of these relapsed. 4 MN pts with residual disease on CT all progressed radiologically.

**Conclusion**: Clinical examination alone is insensitive but when combined with the assessment of CA125 is able to detect a significant majority of disease progression. Although this study supports the NCI recommendation that routine CT scanning is not indicated in the follow-up of ovarian cancer, its role in marker negative patients should be reconsidered. The added value of detecting clinically unexpected findings by CT is subject to ongoing studies.

**1.7**

**MANAGEMENT OF INTERNAL MAMMARY NODES IN SENTINEL NODE BIOPSY**

Professor R E Mansel* – Principal Investigator on behalf of the ALMANAC Trialists Group

Department of Surgery, University of Wales College of Medicine, Cardiff

**Introduction:** Sentinel Node Biopsy (SNB) is a guided technique for determining axillary lymph node status of patients with breast cancer. Whilst it is hoped that SNB will eliminate unnecessary surgery for two thirds of breast cancer patients who currently receive standard axillary treatment, either axillary clearance or axillary sampling, to date there has been no detailed evaluation of this new technique in the form of a large randomised trial. One of the issues raised by the SNB technique is the management of the internal mammary nodes.

The ALMANAC trial (Axillary Lymphatic Mapping Against Axillary Nodal Clearance) is a two staged, multi-centre trial comparing SNB with standard axillary treatment in the management of breast cancer. The first stage, now completed, was an Audit stage where surgeons were evaluated on their ability to successfully perform the technique. The second stage, which is currently ongoing, is comparing SNB with standard axillary treatment in terms of arm and axilla morbidity, health economics and quality of life. The Audit stage data is presented here.

**Method:** In the Audit stage, surgeons performed a sentinel node biopsy before going on to perform their standard axillary procedure, either axillary sampling or axillary clearance. The sentinel node was localised using a combined technique of a radioisotope (Technetium 99m) and blue dye (Patent blue V). In accordance with the ALMANAC protocol the patient was either injected with the radioisotope the day before the operation (40MBq) or on the day of operation (20MBq). This was followed by a static lymphoscintiscan at least three hours from the time of injection. The drainage site was recorded and the number of hot nodes noted.

**Results:** In total 29 surgeons took part in the Audit stage and 803 patients were recruited. Eight patients did not receive a scan due to logistical problems. Data was missing on a further 26 patients leaving 769 cases of analysable data. There were 206 cases (27%) where no drainage site was reported on the scan and 563 cases (73%) where drainage was reported. Axillary drainage was reported in 537(70%) cases and internal mammary drainage in 63(8%). Of the 63 patients, only 22(35%) had lymph nodes removed of which 2 'nodes' proved to be fatty tissue. Only 4 patients had internal mammary nodes that were pathologically positive and in 2 of the patients the axillary nodal status was negative. One patient sustained a pneumothorax and one patient suffered bleeding from the internal mammary artery.

**Conclusion:** Removing the internal mammary nodes remains a difficult routine procedure. Due to the relatively small percentage of patients that have positive internal mammary nodes with negative axillary status (ie have a change in stage), we conclude that routine removal of internal mammary nodes will have a minimal effect on the mortality from breast cancer.

**2.1**

**MOLECULES AND MECHANISMS: THE STORY OF PHORTRESS.**

Tracey D. Bradshaw.

The Pharmacy School, University of Nottingham, Nottingham, NG7 2RD, U.K.

During this presentation, I would like to trace the molecular evolution of Phortress, describing elucidation of the mechanism of action of the enigmatic class of compounds to which it belongs. I hope to illustrate the interaction between chemistry and pharmacology which led to selection of a clinical candidate. The synthesis of polyhydroxylated 2-phenylbenzothiazoles, designed as potential tyrosine kinase inhibitors, yielded 2-(4-aminophenyl)benzothiazole (CJM 126). Intriguingly, CJM 126 exerted pronounced growth inhibition in breast cell lines only, effecting an unusual biphasic dose response. Substitution at position 3 in the phenyl ring with a halogen atom or alkyl moiety enhanced potency in the breast carcinoma panel and extended the spectrum of exquisitely selective antitumour activity to ovarian, renal and colon cell lines ($GI_{50}$<10 nM). *In vivo* evaluation demonstrated the superior antitumour activity of 2-(4-amino-3-methylphenyl)benzothiazole (DF 203). Mechanisms of action were unknown; remarkably similar profiles of antitumour activity within the series failed to COMPARE with any clinical class of chemotherapeutic agent. Crucially, only sensitive cells sequestered DF 203, liberating the *C*-6 hydroxylated metabolite (6OH 203). Planar, hydrophobic aminophenylbenzothiazole analogues are potent ligands for the cytosolic aryl hydrocarbon receptor. CYP1A1 mRNA, activity and protein expression are powerfully induced. NADPH-dependent DF 203-derived covalent binding to recombinant CYP1A1 is reduced by glutathione, suggesting 1A1-dependent formation of (a) reactive electrophilic species. Indeed, 2-(4-aminophenylbenzothiazole-derived DNA adducts are generated in sensitive tumour cells only. Paradoxically however, 6OH 203 is devoid of antitumour activity. This metabolite antagonises cellular uptake of DF 203, covalent binding between CYP1A1 and DF 203, CYP1A1 activity and growth inhibition induced by DF 203. The evidence suggests that CYP1A1-catalysed ring hydroxylation underlies the biphasic dose response. To thwart deactivating metabolism, fluorinated analogues of 2-(4-aminophenyl)benzothiazoles have been synthesised. 2-(4-amino-3-methylphenyl)-5-fluorobenzothiazole (5F 203) elicited a conventional dose response; oxidative metabolism at *C*-6 was eradicated. Superior potency *in vitro* and enhanced efficacy *in vivo* against human breast and ovarian tumour xenografts led to this agent becoming the favoured analogue for clinical consideration. To improve drug bioavailability, the exocyclic primary amine function of lipophilic 2-(4-amino-3-methylphenyl)benzothiazoles has been successfully conjugated to alanine and lysine residues as mono- and dihydrochloride salts respectively yielding water soluble, chemically stable prodrugs. *In vitro*, selective antitumour activity is retained as parent amine is regenerated in the presence of carcinoma cells. *In vivo*, prodrugs rapidly and quantitatively revert to their parent amine. Plasma concentrations of 5F 203 regenerated from the lysylamide prodrug Phortress, sufficient to elicit cytocidal activity against human mammary carcinoma cell lines, persisted > 6h. The growth of breast and ovarian xenograft tumours was significantly retarded by Phortress. CYP1A1 protein expression and DNA adduct formation, selectively induced in sensitive carcinoma cells, has been detected in MCF-7 and IGROV-1 tumours 24 h after treatment of mice with Phortress (20 mg/kg). Phortress will undergo Phase I clinical evaluation in 2002, under the auspices of Cancer Research UK.

© 2002 Cancer Research UK

### 2.2
### THE HISTONE DEACETYLASE INHIBITOR PXD101 INHIBITS THE GROWTH OF HUMAN OVARIAN AND COLON TUMOUR XENOGRAFTS *IN VIVO.*

J.A. Plumb*, R.J. Williams[1], P.W. Finn[1], M.J. Bandara[1], M.R. Romero[1], C.J. Watkins[1]. N.B. La Thangue[1] and R. Brown.
Department of Medical Oncology, University of Glasgow, Cancer Research UK Beatson Laboratories, Glasgow, G61 1BD and [1]Prolifix Ltd, Abingdon, OX14 4RY.

Histone deacetylation has a central role in the control of gene expression, including transcriptional repression of tumour suppressor genes. Treatment of tumour cells with inhibitors of the enzyme histone deacetylase (HDAC) results in gene activation, growth suppression and induction of differentiation or apoptosis. PXD101 is a novel hydroxamate type inhibitor of HDAC that inhibits HDAC activity in HeLa cell extracts with an $IC_{50}$ in the range 20-40nM. PXD101 inhibits the growth *in vitro* of a range of tumour cell lines with $IC_{50}$s in the range 0.3 - 5.7μM as determined by an MTT assay. The pattern of sensitivity *in vitro* observed for PXD101 in the cell lines is distinct from that observed for the HDAC inhibitor oxamflatin and for the DNA damaging cytotoxic cisplatin. In contrast, there is a clear correlation between sensitivity to oxamflatin and cisplatin ($r^2$ = 0.7). A concentration-dependent (0.2 - 5μM) increase in acetylation of histones H3 and H4 is observed in tumour cell lines including colon, lung, breast and prostate. Furthermore, the level of acetylation observed at 0.2μM correlates with the sensitivity of the cell line. Incubation of the human ovarian cell line A2780 with PXD101 (1μM) results in acetylation of H3 & H4 within 30 minutes that is retained for up to 36 hours with continuous incubation. For oxamflatin, acetylation is maintained for only 6 to 8 hours. Following removal of PXD101, acetylation is maintained for about 30 minutes but is markedly decreased after 1 hour. PXD101 (2μM) induces apoptosis in A2780 and in the colon cell line HCT116 as characterised by PARP cleavage after treatment for 24 hours.
Treatment of nude mice bearing A2780 tumour xenografts with PXD101 (10 - 80mg/kg/day intraperitoneally) daily for 7 days causes a significant dose dependent growth delay with no obvious signs of toxicity to the mice. Growth delay is also observed for xenografts of the cisplatin resistant derivative of A2780 (A2780/cp70) and for HT29 and HCT116 colon xenografts. A marked increase in acetylation of H4 is observed in blood, tumour and skin of mice 3 hours after treatment with PXD101 at 20 and 40mg/kg.
Thus the histone deacetylase inhibitor PXD101 inhibits growth and induces apoptosis in human tumour cells. The inhibition of growth of human tumour xenografts in mice, with no obvious toxicity, suggests that PXD101 has potential as a novel anti-tumour agent. Furthermore, measurement of histone acetylation in blood or skin could provide a suitable pharmacodynamic endpoint to monitor drug activity.

### 2.3
### ANTISENSE DOWNREGULATION OF GENE EXPRESSION IN CHRONIC MYELOID LEUKEMIA; DELIVERY COUNTS.

O Connor L[1]*., Gately K[1]., McElwaine S[1]., McCann SR[1]., Cotter T[2]., Hollywood D[1]., Lawler M[1]. Depts [1]Haematology St James's Hospital and Trinity College Dublin, Ireland, [2] Biochemistry, University College Cork Ireland.

Chronic Myelogenous Leukemia(CML) is a myeloproliferative disorder characterised by a disease specific marker t(9,22) which gives rise to a leukaemia specific RNA *(bcr-abl)* involving either a fusion of exon 2 of *bcr* and exon 2 of *abl* (b2a2) or exon 3 of *bcr* and exon 2 of *abl* (b3a2). Both RNAs encode a tyrosine kinase (P210) that makes CML cells resistant to conventional chemotherapy. *Bcr-abl* could serve as a target for molecular directed therapies. Antisense oligonucleotides (ODNs) are short stretches of DNA, approximately 18-20bp in length, with a sequence complementary to a specific mRNA. Theoretically, they provide a mechanism for inhibition of expression of disease causing genes while leaving the expression of non-targeted genes unaffected. Antisense ODNs directed against either the b2a2 or b3a2 breakpoints to downregulate *bcr-abl* expression were initially tested in material from bone marrow donors using both short term (CFU-GM) and long term (CAFC) culture assays. These assays indicated that antisense ODNs display minimum toxicities against normal haemopoietic stem cells, a critical requirement before proceeding to examine the effects on malignant cells. Testing of fluorescently labelled antisense ODNs (by FACs and fluorescent microscopy) indicated that they can penetrate CML cell lines (K562, KYO1, LAMA 84 and BV 173) and uptake was consistent with fluid phase endocytosis. Testing of CML cell lines by western blot analysis indicated downregulation of P210. However in all cell lines tested, downregulation of expression of P210 was variable (10-40%) and transient (<24 hours). Primary cells from CML patients (n=4) were treated with 10uM antisense for 48 hours and 2/4 exhibited a reduction in CFU-GM colony number (36 – 40%) indicating a specific anti-proliferative effect in CML cells. In order to improve downregulation of P210 and enhance antileukemic effects, we examined the kinetics of uptake of antisense ODNs using either carrier systems or approaches that permeablise the cell to antisense delivery. A critical component of the antisense approach is its ability to traverse the cell membrane whose lipid bilayer naturally repels the charged antisense molecule. Thus successful antisense approaches require an efficient method of cellular delivery. We have tested a number of carrier systems including different liposome and dendrimer formulations which while achieving improved uptake (as judged by fluorescent and confocal microscopy of fluorescently labelled ODNs) also exhibit significant toxicity to normal cells (10-30%). Streptolysin-O(SL-O) is a bacterial toxin, which forms pores in the cell membranes of eukaryotic cells by binding to cholesterol. Following binding and polymerisation therein, resealing of the pores is then induced by addition of cholesterol containing FCS. SL-O was the most efficient method of delivery of oligonucleotides into the nucleus and cytoplasm, giving uptake > 95% in the 4 CML cell lines tested and cell death was minimal. Real time PCR using TaqMan technology indicated 2-3 log reduction in *bcr-abl* transcripts at 24-48 hours. Thus appropriate choice of delivery system can greatly enhance the antisense effect in CML cells. Future approaches include the selection of antisense ODNs with superior hybridisation properties using either morphilino linkages or microarray technology.

### 2.4
### HA14-1, A SMALL MOLECULE BCL-2 ANTAGONIST: CHEMOSENSITZATION AND PHARMACODYNAMICS IN LEUKAEMIA

Dean A. Fennell*, Margherita Corbo, Arben Pallaska, Finbarr E. Cotter
Department of Experimental Haematology, Barts and The Royal London, Queen Mary's School of Medicine and Dentistry, London, E1 2AD

Induction of apoptosis is a mitochondrial final common pathway underlying the efficacy of cytotoxic therapy for leukaemia, and its inhibition confers an important mode of chemoresistance. Bcl-2 is a mitochondrial protein that heterodimerizes with, and blocks the action of death agonist homologues Bax and Bak, essential for chemotherapy induced apoptosis. HA14-1 is a first generation, low molecular weight, organic Bak BH3 peptide mimetic that binds Bcl-2 and Bcl-$X_L$, with micromolar affinity, and blocks death agonist heterodimerization. Putative synergistic interactions between HA14-1 and chemotherapy were therefore investigated quantitatively in HL60, BV173, and Bcl-2 transfected K652 leukaemia cell lines. Tolerance distributions for HA14-1, VP16 and ara-C induced mitochondrial depolarisation ($\Delta\Psi_m$ collapse, measured using the amphipathic probe $DiOC_6(3)$ and propidium iodide), and apoptosis (determined morphologically, and by ZVAD.fmk inhibitable loss of plasma membrane lipid packing using MC540 and 7AAD), were measured at single cell resolution using flow cytometry, then fitted by modified Hill equation using maximum likelihood non-linear regression. Fixed ratio combinations of HA14-1 with VP16 or Ara-C yielded concave-up (synergistic) isobolograms. Quantitative interaction analysis by the Chou-Talalay method demonstrated significant synergy (combination index < 0.5) in the ED25 and ED75 dose range following exposure for 48 hours. The magnitude of the measured combination index was time dependent, synergy increasing with the duration of exposure. HA14-1 exhibited significant schedule dependence in all of the cell lines studied, with greater cytotoxicity being observed for HA14-1 administration pre-chemotherapy, in contrast to HA14-1 administration following VP16. Mitochondrial dysfunction and apoptosis were induced to a similar degree in Bcl-2 transfected K562 cells (that constitutively express Bcl-$X_L$) compared with vector only transfected cells. HA14-1 induced $\Delta\Psi_m$ collapse occurred within an hour at 25 micromolar concentration, was irreversible following 30 minutes exposure, and was not suppressed by the pan-caspase inhibitor, Z-VAD.fmk. In the functional p53 expressing cell line MOLT-4, HA14-1- induced cell death was not prevented by the p53 inhibitor pififthrin in contrast to VP16 induced cell death, supporting a p53 independent mechanism of cell death. Using real-time flow cytometry, rapid oxidation of the redox sensitive probe

© 2002 Cancer Research UK

dihydroethidium was observed within 15 minutes following administration of HA14-1, that was not prevented by the superoxide dismutase mimetic MnTBAP, nor the mitochondrial permeability transition pore complex inhibitor, cyclosporin A. In summary, HA14-1 is a novel small molecule that interacts with Bcl-2 or Bcl-$X_L$, and sensitises cells to chemotherapy induced cell death. This represents a new lead compound for the development of future therapeutic strategies for reversing chemoresistance in leukaemia.

### 2.5
### CAPTOPRIL INHIBITS THE MATRIX METALLOPROTEINASES: MMP-2 AND MMP-9

RN Williams[1]*, RA Dean[1], SL Parsons[3], BJ Rowlands[2], SA Watson[1]
[1]Academic Unit of Cancer Studies, [2]Dept. of Surgery, QMC, University Hospital, Nottingham, NG7 2UH; [3]Dept. of Surgery, City Hospital, Nottingham

**Background:** The matrix metalloproteinases (MMPs) are a family of zinc dependent endopeptidases capable of degrading components of the extra-cellular matrix (ECM). The activity of these enzymes is crucial for lysis of the ECM that occurs during tumour angiogenesis, tumour growth and metastasis. Angiotensin Converting Enzyme (ACE) inhibitors, such as Captopril, inhibit the activity of the zinc dependent metallopeptidase enzyme ACE, by binding the zinc moiety of the enzymes active site. The aim of this study was to determine whether the ACE inhibitor Captopril could inhibit the activity of other zinc dependent enzymes involved in malignant progression, namely MMP-2 and -9.
**Methods:** Gelatin zymography was used to investigate inhibition of the activities of MMP-2 and MMP-9 by Captopril. Conditioned media from the human fibrosarcoma cell line HT1080, (known to secrete both MMP-2 and MMP-9 in large quantities) was used for zymography following protein determination. Captopril was added to the developing buffer for the overnight incubation in a range of concentrations. Subsequently, a known quantity of HT1080 cells was incubated over night with a fixed volume of serum free medium containing differing concentrations of Captopril. Zymography was performed without protein determination and the quantity of cells re-counted on removal of the media.
**Results:** Captopril inhibited the activity of both MMP-2 and MMP-9 in a dose dependent fashion when added to zymography developing buffer. MMP-9 (92kDa) was inhibited to 70.7% (p=0.038), 64.8% (p=0.023), and 46.9% (p=0.002) of control values by 500μM, 1mM and 2.5mM Captopril respectively. Pro-MMP-2 (72kDa) was inhibited to 72.9% (p=0.007), 72.3% (p=0.046), 55.8% (p=0.001), 40.9% (p=0.002) and 27.2% (p<0.001) by 250μM, 500μM, 1mM, 2.5mM and 5mM Captopril respectively. Active MMP-2 (62kDa) was inhibited to 23.4% (p<0.001) and 9.3% (p<0.001) by 250μM and 500μM Captopril respectively. The addition of 5mM Captopril to cell culture of HT1080 produced inhibition of MMP-9 activity to 65% (p=0.017) of control values whilst MMP-2 activity was 66.1% (p=0.016) for pro-MMP-2 and 75% (p=0.015) of control values for active MMP-2. 5mM Captopril inhibited the proliferation of HT1080 when added to cell culture. The population of cells treated with 5mM Captopril was only 84% (p=0.047) of the untreated control population.
**Conclusions:** These data demonstrate that the ACE inhibitor Captopril is capable of inhibiting MMP-2 and -9. Inhibition of MMP activity by Captopril added to zymogram developing buffer suggests that Captopril inhibits MMPs by binding their active site. The inhibition of MMP activity produced by the addition of Captopril to cell culture is greater than its inhibitory effect on cell proliferation. This suggests that Captopril may inhibit other cellular pathways and that the reduction in MMP activity is not only a reflection of the reduction in cell population.

### 2.6
### INHIBITORY EFFECT OF NOVEL NON-PEPTIDE CHOLECYSTOKININ ANTAGONISTS ON GROWTH OF HUMAN GLIOMA CELLS IN CULTURE

Eftychia Oikonomou*, Eric Lattmann and Eric F Adams, Pharmaceutical Sciences Research Institute, Aston University, Aston Triangle, Birmingham B4 7ET, UK.

Gliomas are very serious brain tumours with life expectancy after diagnosis averaging only nine months. Their inaccessible location and / or adherence to surrounding structures often hinder complete removal of these tumours by neurosurgical procedures. It is therefore desirable to develop improved adjuvant medical therapies which are able to strongly inhibit or eliminate residual tumour growth thereby reducing the chances of recurrence. Previous studies have demonstrated that the gut-brain peptide, cholecystokinin (CCK), is able to powerfully stimulate growth of rat glioma cells *in-vitro* (1). It might therefore be anticipated that blockade of CCK receptors will have an inhibitory effect on glioma cell mitosis. We have developed a number of non-peptide asperlicin CCK antagonists and examined their effects on growth of human glioma cells in culture. Cells were seeded at a density of 1 x $10^5$ per flask and grown in the presence of varying doses (0-20 μmol / L) of two of these novel compounds, HSH and 7.4.5, for up to two weeks. After this period, cells were removed from the flasks by trypsinisation and their number determined by means of a Coulter Counter. Both compounds strongly inhibited growth of the human glioma cells by up to 80% in a dose-dependent manner. In addition, the stimulatory effects of epidermal growth factor on glioma cell growth was completely abolished by co-incubation with each of the CCK antagonists. A time course study revealed that these inhibitory effects remained consistent for at least two weeks in culture. The results suggest that CCK antagonists are able to inhibit human glioma cell growth. Because of their non-peptide nature, it may be possible to further develop these compounds as orally active anti-glioma drugs.

1) Kaufmann R et al (1998) Protein kinase C is involved in cholecystokinin octapeptide-induced proliferative action in rat glioma cells. Neuropeptides 32: 185-189.

### 2.7
### ACTIVITY OF CRE-DECOY OLIGONUCLEOTIDES IN COLORECTAL CANCER CELL LINES.

WM Liu*, KA Scott, SP Joel and DJ Propper.
Dept Medical Oncology, St Bartholomew's Hospital, London.

The cAMP response element (CRE) consensus sequence directs the transcription of a wide range of genes. A 24-mer ss oligonucleotide sequence has been shown to compete with these sequences for binding transcription factors and therefore interferes with cAMP-induced gene transcription. We have examined the cytotoxic effect of this CRE-decoy oligonucleotide (CDO) alone and in combination with a range of chemotherapeutic drugs in colorectal cancer cell lines. Cell viabilities and cell cycle distributions were assessed daily throughout a 4-day treatment schedule, and in cells treated for 4-days followed by drug-free medium. CDO was an effective cytotoxic agent when used alone in HCT116 and GEO cell lines ($IC_{50}$: 0.29 μM and 0.36 μM respectively), but ineffective in SW620 cells (viability > 85% at all time points with CDO up to 1.6 μM). Control oligonucleotides had no effect of cell proliferation and viability. Loss of viability in the sensitive cells was associated with increases in p21$^{waf1}$ and bax protein expression, but with no change in bcl-2 levels. Flow cytometric analysis indicated increased apoptosis (A) with concomitant reduction in cells in the G1-phase of the cell cycle (day 4 %A and %G1: 19.8 ± 1.0 and 26.1 ± 1.6 vs. 2.5 ± 1.0 and 45.6 ± 2.5 respectively in control cells; all p<0.001). The combination of CDO with existing chemotherapy resulted in additive cell kill. However, there were synergistic reductions in total cell number, which were small but consistent (the table shows results from the cultures with each drug at $IC_{25}$ in HCT116).

| cells (x10$^6$/ml) | 5-fluorouracil | Oxaliplatin | Mitomycin | Etoposide |
|---|---|---|---|---|
| **Observed** | -20.9 ± 3.9 | -18.1 ± 4.6 | -17.1 ± 2.8 | -9.3 ± 1.7 |
| **Estimated** | -16.4 ± 4.6* | -13.9 ± 7.4* | -15.4 ± 2.9 | -6.6 ± 2.0* |

*Estimated values are the calculated sum of the reduction in cell numbers from separate CDO and drug cultures. *p<0.01 vs. observed value.*

Data also indicated that the re-culture of CDO pre-treated cells in drug-free medium resulted in continued cell kill and apoptosis even in the absence of drug. Notably, this extent of cell kill was actually greater than that seen in the cultures maintained in CDO. This was demonstrated most clearly in GEO cells where there was nearly a two-fold increase in apoptosis (%A: 4-days CDO + 4-days in drug-free medium: 31.3 ± 1.6% vs. 19.8 ± 1.0% in cells treated with CDO for 8-days; p<0.001). These results confirm the cytotoxic effect of CDOs *in vitro*, and also highlight the schedule-dependent nature of its activity. Studies are on going to elucidate the relationship between CDO cytotoxicity and cell cycle regulation.

© 2002 Cancer Research UK 

3.1

**TUBULAR CARCINOMA OF THE BREAST: IS THE LONG TERM SURVIVAL ASSURED?**

A Bashorun*[1], N Beechey-Newman[1], K Ryder[2] and AE Young[1]
1.Breast Unit, Guy's Hospital, London SE1 9RT.UK
2.ICRF , Guys Hospital, London SE1 9RT. UK.

**Aims** Pure tubular invasive carcinoma (TC) of the breast (Grade I and more than 90% tubular formation) is thought to have favourable prognosis in treated patients observed over short and intermediate periods of 5-10 years. There is, however, little data relating to more extended follow up. This study aimed to review the long term (20-25 years) survival of patients with pure tubular carcinoma and to investigate whether this lesion does confer a better survival compared with the control group of other commoner grade I invasive ductal carcinomas of no specified type, (NST).

**Materials and Methods** Case records from 1975 – 2000 were obtained for all grade I invasive ductal carcinomas (NST and TC) treated at Guy's Hospital London.

There were 73 patients with pure TC and 420 grade I (NST). These cohorts were of similar age (TC median 52 years (35-83); G1 (NST) median 55.1 years (27-88). The mean follow up was 9.7 years. All patients had tumourectomy and complete axillary dissection. Kaplan-Meier survival curves were compiled and statistical analysis was carried out using the Log Rank method.

**Findings** There was no correlation between histological type and age (p=0.102) or nodal status (p=0.205). In the TC group survival was significantly better in patients without lymph node metastasis (p=0.0412). No significant statistical differences in long-term survival of patients with TC and those with G1 (NST) were identified (P = 0.487). Moreover the similarity in overall survival remained after sub-division into node negative (p= 0.23) and node positive (p=0.70) groups.

**Conclusion** Although pure tubular carcinoma has been thought to be a favourable sub-type of breast cancer, it is concluded that its overall long term survival is no better than that of the commoner grade I invasive carcinoma of non-specific type. Therefore tubular carcinoma should continue to be regarded as potentially life threatening in the longer term and accordingly should not merit an attenuated treatment or follow up protocol.

3.2

**WEIGHT LOSS AND ANAEMIA ARE POOR PROGNOSTIC FACTORS FOR PATIENTS WITH LUNG CANCER RECEIVING CHEMOTHERAPY – WILL MODIFICATION OF THESE PROGNOSTIC FACTORS BECOME A NEW STANDARD OF CARE?**

Paul J Ross*, Justin S Waters, Sue Ashley, Kathy Priest, Alison Norton, Tim Eisen, Ian E Smith, Mary ER O'Brien. Lung Unit, Royal Marsden Hospital, Sutton, Surrey, SM2 5PT

This study aimed to examine whether weight loss at presentation influences outcome in patients receiving chemotherapy for small cell (SCLC) and non-small cell lung cancer (NSCLC). A multivariate analysis was performed on data collected prospectively from 1994-2001. Data were available for histology, stage, performance status, response, toxicity, progression-free and overall survival. Weight loss at presentation was reported by 59% of 290 patients with SCLC and 58% of 418 patients with NSCLC. Patients with weight loss and NSCLC more frequently failed to complete at least 3 cycles of chemotherapy than patients without weight loss (p=0.003). Anaemia as a toxicity occurred significantly more frequently in patients with NSCLC and weight loss (p=0.0003). No differences in other toxicities were observed. NSCLC patients with weight loss had more symptoms at presentation (p<0.0001) and were less likely to have a symptomatic response (p=0.001). In addition, there was a trend towards fewer symptomatic responses in patients with weight loss and SCLC (p=0.06). Overall survival was reduced in patients with weight loss and SCLC (8 months v 11 months; p=0.0003) and NSCLC (6 months v 9 months; p<0.0001). Similarly, progression-free survival was shorter in patients with weight loss for both SCLC (6 months v 7 months; p=0.004) and NSCLC (4 months v 6 months; p=0.01). In multivariate analyses performance status and stage were the most important predictors of progression-free and overall survival. However, weight loss remained an independent predictor of shorter overall survival for patients with SCLC (p=0.003, relative risk=1.5) and NSCLC (p=0.009, relative risk=1.33) and an independent predictor of shorter progression-free survival in patients with SCLC (p=0.01, relative risk=1.43). Patients with NSCLC whose weight stabilised during chemotherapy had better overall survival than patients who continued to lose weight (p=0.0004).

Weight loss is an early symptom of lung cancer and predicts for toxicity from treatment, in particular anaemia, and shorter survival. We had previously observed that 86% of patients receiving chemotherapy for SCLC and NSCLC experienced a nadir haemoglobin < 12g/dL (Waters et al, 2002. J Clin Oncol; 20: 601). In addition, survival was shorter for patients with a nadir haemoglobin <12 g/dL (9 months) compared to those with haemoglobin ≥ 12 g/dL (12 months; p=0.001). We conclude that a trial evaluating nutritional supplements combined with correction of anaemia in patients receiving chemotherapy should be considered. Such interventions may improve quality of life and possibly survival in lung cancer patients and may lead to a new term 'total supportive care'.

3.3

**IMPACT OF HAEMOGLOBIN LEVEL ON TREATMENT RESPONSE IN PATIENTS WITH ANAL CARCINOMA**

G Hutchison*,[1] AG Renehan,[1] E Levine,[2] R D James,[2,3] ST O'Dwyer.[1]
Departments of [1]Surgery and [2]Clinical Oncology, Christie Hospital, Manchester, UK.
[3]Present address: Kent Cancer Centre, Maidstone, UK.

**Introduction:** Radiotherapy ± chemotherapy is the mainstay of treatment for anal carcinoma but local recurrence rates requiring salvage surgery remain high (30-40%). In more-radiocontrollable tumours an increase in the hypoxic cell fraction is associated with treatment-time anaemia and poor outcome (1,2). We have evaluated this in patients with anal cancer.

**Methods:** Between 1988 and 1998 at the in a single institute, 206 patients with anal squamous cell carcinoma were treated with curative intent using radiotherapy alone(RT: n=113) or chemoradiotherapy (CRT: n=93). Haemoglobin(Hb) levels before and during treatment were collected, and the nadir (lowest values) Hb calculated. Treatment failure was determined histologically within twelve months of initial therapy. Univariate (Kaplan-Meier) and multivariate (Cox proportional hazard) analyses were used with Hb levels treated as categorical variables (cut-point: medians by gender).

**Results:** Persistent local disease occurred in 37% of cases. There was a non-significant trend toward decreasing Hb with increasing stage (T1: 12.8g/dl; T4 11.8g/dl, K-W: P=0.11). In RT patients, nadir Hb values below the median (males: 12.7g/dl; females: 11.9g/dl) were highly predictive for failure (Kaplan-Meier, P<0.001). This remained significant after adjustments for stage, age and sex (hazard ratio: 6.20,2.86,1.32, CI 95%). No association was evident with CRT.

**Conclusion:** In patients with anal squamous cell carcinomas, nadir Hb level is predictive of response to RT alone but not chemoradiotherapy. These findings justify further studies into the role of anaemia in tumour radio-resistance.

(1) Hockel M et al. Cancer Res 1999;59:4525-8.
(2) Henke M et al. Int J Radiat Oncol Biol Phys 2000;48:339-45.

3.4

**hTERT GENE EXPRESSION IS REGULATED BY ALTERNATIVE SPLICING OF mRNA DURING THE COURSE OF CML, AND TELOMERE LOSS AT DIAGNOSIS IS ASSOCIATED WITH EARLY PROGRESSION.**

MW Drummond*[1], S Hoare[2], MJ Alcorn[1], A Monaghan[2], S Graham[1], WN Keith[2], TL Holyoake[1]. [1]ATMU, University of Glasgow, UK; [2]Beatson Institute, Garscube, UK.

***Background:*** Chronic myeloid leukaemia (CML) is characterised by myeloid expansion, subsequent to acquisition of the Philadelphia chromosome (Ph). Telomere shortening has been demonstrated in peripheral blood leucocytes (PBL) of CML patients. Short telomeres in chronic phase disease (CP) have been shown to predict for early disease progression, perhaps due to early cell 'crisis' and acquisition of further mutations. The regulation and role of telomerase (in particular the catalytic component, hTERT) during early disease remains poorly understood. We prospectively studied newly diagnosed CML patients with regard to degree of telomere loss and outcome, and examined levels of hTERT mRNA and its splice-variants in samples from all stages of CML. ***Methods***: Flow-FISH telomere measurement of Ph-

© 2002 Cancer Research UK

© 2002 Cancer Research UK

